# Maternal immunity and African swine fever virus: understanding the limits of passive protection

**DOI:** 10.3389/fimmu.2025.1593820

**Published:** 2025-07-25

**Authors:** Virginia Friedrichs, Mathias Streitz, Martin Beer, Sandra Blome, Alexander Schäfer

**Affiliations:** ^1^ Institute of Diagnostic Virology, Friedrich-Loeffler-Institut, Greifswald – Island of Riems, Germany; ^2^ Department of Experimental Animal Facilities and Biorisk Management, Friedrich-Loeffler-Institut, Greifswald – Island of Riems, Germany

**Keywords:** African swine fever, African swine fever virus, antibodies, maternal immunity, humoral immune response

## Abstract

African swine fever (ASF) is an often-fatal disease impacting domestic and wild pigs world-wide. Understanding the role of maternal immunity in ASF pathogenesis is crucial for effective control. This study characterized kinetics and protective potential of maternal immunity against ASF virus (ASFV) in neonatal piglets. Two times ten sows were inoculated with the moderately virulent ASFV strain ‘*Estonia2014*’, all developed typical ASF signs and viraemia; five animals recovered. The offspring of two recovered sows (*n* = 24) were sampled weekly to monitor maternal ASFV-specific antibody kinetics. The offspring of two other sows, in addition to piglets of an ASFV-naïve sow, were challenged oro-nasally with the highly virulent ASFV strain ‘*Armenia2008*’ on the seventh day of life. To evaluate the impact of ASFV-specific antibodies without ASFV-specific T cells, five piglets from the naïve sow received purified, concentrated immunoglobulins from ASFV-immune pigs via serum transfer prior to challenge infection. All naïve piglets (*n* = 12), regardless of immunoglobulin transfer, reached the humane endpoint 6 days post inoculation (dpi). Piglets of immune sows began displaying clinical signs 5 dpi, and all either succumbed or reached the humane endpoint by 9 dpi (*n* = 27). Serology confirmed antibodies against ASFV (p32, p72) in all piglets of immune sows. Antibody titers in unchallenged piglets remained stable for at least 60 days after birth. In challenged piglets, those of immune sows were initially seropositive but mostly seronegative after challenge, indicating antibody consumption. Passively transferred antibodies were also depleted after challenge. In conclusion, passively acquired immunity, whether through immunoglobulin transfer (antibodies) or colostrum (antibodies and lymphocytes), is insufficient to safeguard neonatal pigs from lethal infection with highly virulent ASFV.

## 1 Introduction

African Swine Fever (ASF) is recognized as one of the most complex viral diseases affecting the whole pig production and husbandry sector, imposing significant socio-economic burdens and public health concerns in affected countries (1, 2). The transboundary nature of ASF, exacerbated by the lack of an effective vaccine or treatment, underscores its importance as a disease of global concern (3). The hemorrhagic disease is caused by the ASF virus (ASFV), a double-stranded and large DNA virus with complex immune evasion mechanisms (4). Infected pigs (domestic pigs and Eurasian wild suids) typically exhibit severe clinical signs, often leading to very high lethality rates (5). Over the past 15 years, ASF has shown unprecedented spread across domestic and wild pig populations globally, classifying it as a panzootic threat to both agriculture and wildlife (6).

The spread of ASF in wild boar populations across various countries necessitates the establishment of intensive monitoring systems. Considerations of long-term consequences of sexually mature females surviving an ASFV infection play a crucial role. Those females hold the potential to pass on ASFV-specific maternal immunity, especially antibodies and reactive T cells, to their offspring. This inevitably results in seropositive animals that passively acquired immunity but did not survive an ASFV infection by themselves. Therefore, maternal immunity should be considered in the establishment of monitoring/surveillance schemes and vaccination strategies. Recent advancements and initial successes in recent years have brought hope for controlling the disease (7–9); however, the unclear role of maternal immunity in offspring protection presents considerable challenges for vaccine deployment and effectivity. As the blood-placental barrier is impassable in pigs, piglets are born immunologically naïve and are thus exposed to a high risk of infection. As a countermeasure, piglets receive high amounts of antibodies and lymphocytes through uptake of the first milk by their mothers, so-called colostrum, providing passive immunity immediately after birth. This provides temporary protection by transferring humoral and cellular maternal immunity and, thus, might also affect the outcome of an ASFV infection during this time (10). However, passive immunity’s waning over time renders piglets vulnerable later on. Moreover, maternal immunity might interfere with the development of an active immune response in piglets receiving potential ASFV vaccines (10), similar to observations with live attenuated vaccines against classical swine fever (CSF), where maternal antibodies provide partial protection, potentially masking clinical signs but failing to prevent disease transmission completely (11–13). Comparable interference of maternal immunity with vaccination has also been described in multiple other species (14–21). Therefore, determination of the correct time for vaccination in young animals is crucial to overcome the susceptibility gap between maternal immunity and individual immune responses in the offspring.

Additionally, maternal immunity is affected by strain-specific virulence in field virus infections. While only attenuated strains allow for the development of a protective immune response, the outcome of such infections might differ between pregnant and non-pregnant females, boars or juvenile animals. Only one example exists where maternal immunity was discussed as one major cause for increased survival rates and seroprevalence in a population burdened by a highly virulent ASFV strain: Sardinia (2, 22).

The present study aimed to close current knowledge gaps in understanding the role of passive immunity for ASF disease dynamics. We examined how immunity generated during an immune response to a moderately virulent ASF strain–which allows for survival of sows–was transferred through colostrum to piglets. Furthermore, possible protective effects of this maternal immunity were assessed by using a highly virulent challenge infection of the piglets with the ASFV type circulating since many years now globally. Additionally, kinetics and duration of maternal antibodies in piglets were analyzed in more detail.

## 2 Materials and methods

### 2.1 Experimental design

All animals were kept in high-containment facilities at the Friedrich-Loeffler-Institut (FLI). In total, the trial included 22 young sows, two indicator boars and ultimately 61 piglets. Prior to transfer to the FLI, all animals were tested negative for common swine pathogens (e.g., CSFV, ASFV, and PRRSV). The animals originated from a commercial pig breeding facility (Bundes Hybrid Zucht Programm, BHZP) to ensure acquisition of high-standard and high-hygiene breeding sows for this trial. The animal experiment was performed in accordance with current regulations for animal welfare in Germany. Ethical approval was obtained from the responsible authority (Landesamt für Landwirtschaft, Lebensmittelsicherheit und Fischerei Mecklenburg-Vorpommern [LALLF M-V]) under file reference 7221.3-1-011/23.

**Figure 1:**
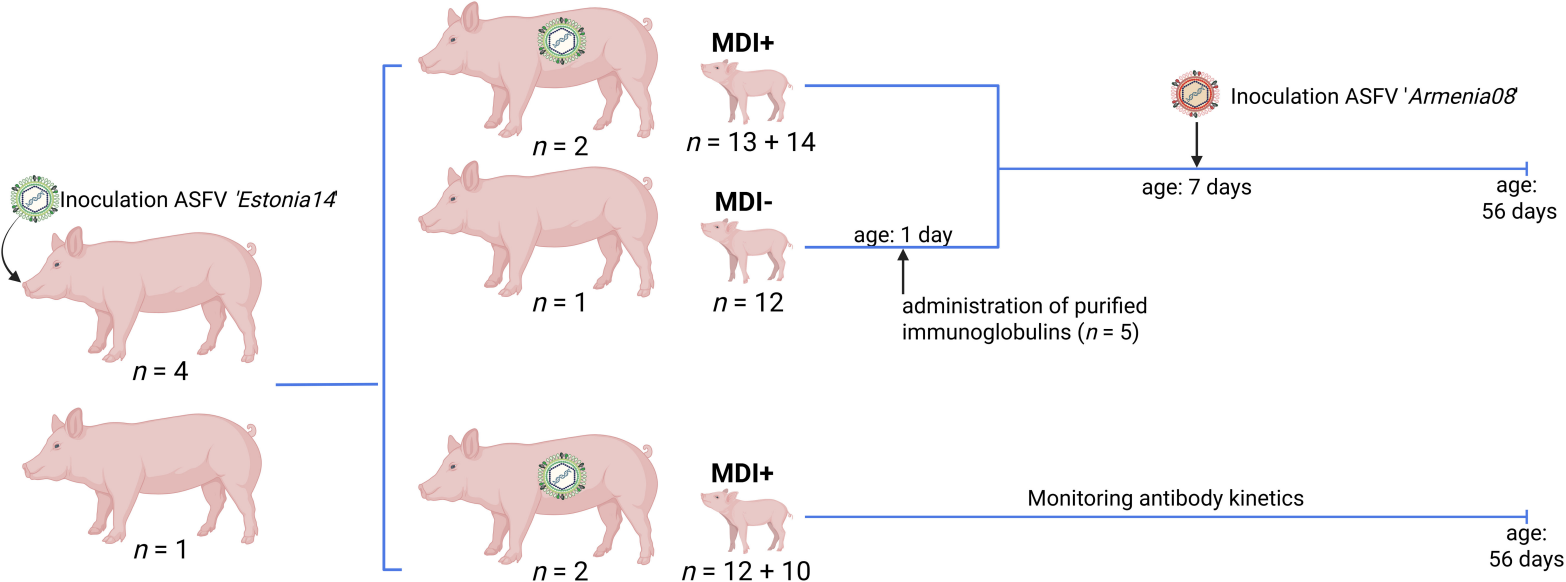
Graphical representation of the animal study design. Actual numbers of all sows and piglets per study section are indicated. MDI, maternal-derived immunity. Generated with BioRender.

Initially, 12 sows were acquired and divided into three pens, holding five, five, and two sows. The group containing two sows was kept in a separate stable as uninfected controls. Additionally, two boars were also kept in a separate stable unit as indicators for estrus. All animals received a unique ear tag to ensure definite identification of individuals at all times: #486, #487 (boars), #872, #939 (control sows), #4, #11, #12, #17, #20, #270, #946, #947, #984, #986 (inoculated sows). After a one-week acclimatization phase, ten sows were oro-nasally inoculated with a spleen suspension containing ~10^4.5^ hemadsorbing units 50% (HAD_50_) per ml of the moderately virulent ASFV strain ‘*Estonia2014’* (23) (**Figure 1**). All pigs received 2 ml of the suspension in each nostril and 6 ml into the mouth. Starting at 0 dpi, rectal temperatures and clinical scores were assessed daily. Successful infection was ascertained 7 dpi with testing blood samples by qPCR. The clinical score [ (24) with slight modifications] monitored changes in behavior and appearance assigning up to three score points per parameter (with increasing severity). A cumulative score of 15 score points was set as humane endpoint. The cumulative score results from the evaluation of various parameters to assess changes attributed to ASFV infection. Evaluated parameters are liveliness, gait, bearing, skin/eyes, feed intake/appetite and faeces, and breathing. Moreover, animals were euthanized that showed unacceptable suffering, e.g. paralysis of the hind legs, seizures or other severe neurological indications. Euthanasia (upon reaching the humane endpoint or at the end of the trial) was executed under deep anesthesia. The anesthesia was induced using one of the following combinations: Zoletil^®^ 100 (Tiletamine/Zolazepam): 3.3 mg/kg, Ketamine 10%: 1–3 ml per 10 kg body weight (equivalent to 10–30 mg/kg), Xylazine (Rompun): 1.6 mg/kg or Azaperone: 0.5 ml per 10 kg body weight, Ketamine 10%: 1–3 ml per 10 kg body weight (equivalent to 10–30 mg/kg). Euthanasia was subsequently carried out by administration of pentobarbital (Release^®^) or by exsanguination (blood withdrawal), ensuring a state of unconsciousness and insensibility and avoiding pain and distress.

Since 60% lethality after inoculation was unexpectedly high given the strain used for inoculation, with only three sows left at 24 dpi, another ten sows were included in the trial at 24 dpi (#316, #366, #393, #394, #396, #297, #298, #355, #373, #380). These ten sows were separated into groups of five and comingled with the animals that survived the inoculation. These sows were infected by their pen-mates within several days after arrival, again confirmed by qPCR 7 days following comingling. Lethality in this group was 80%.

Ultimately, five sows (#17, #297, #394, #984, #986) survived the ASFV infection and were, together with both control sows (#872, #939), subjected to synchronization. Since previous trials showed that sows that get infected with ASFV during early pregnancy will abort in ~90% of cases due to development of high fever (25), synchronization was initiated when all animals tested negative for ASFV in blood by qPCR (80 dpi). While the addition of sows introduced some degree of heterogeneity, it was inevitable for achieving the study’s goals. To reduce potential effects, sows belonged to the same breed and were purchased from the same commercial pig breeding facility. Moreover, since we waited until all animals tested negative for ASFV, there was sufficient time to mount proper immune responses.

For synchronization, all sows received 5 ml Regumate^®^ orally (20 mg Altrenogest, MSD) on 18 consecutive days in the early morning. On the next day, all sows received 2 ml Estrumate^®^ intramuscularly (0.175 mg cloprostenol, MSD) for estrus induction. The following day, all sows received 5 ml Pregmagon^®^ intramuscularly (1000 IE pregnant mare serum gonadotropin, Ceva).

Indicator boars were led to the sows twice a day to assess readiness for artificial insemination. Commercial boar semen (breed db. Siegfried) was acquired from BHZP (Bösewig, Germany). All sows were artificially inseminated at least twice. All sows received an ultrasound examination 30 and 35 days after insemination to verify successful implantation of embryos. One control sow (#939) did not implant embryos.

Since one sow (#394) aborted late in pregnancy with an unknown cause (99^th^ day of pregnancy, all embryos tested negative for ASFV genome), only five sows farrowed after ~114 days of pregnancy. All piglets received a unique ear tag on the 3^rd^ day of life, to ensure accurate identification of individuals (see **Supplementary Table S1**). Additionally, all piglets received 2 ml Ursoferran (200 mg iron-ion solution, WDT) subcutaneously (knee fold) on the same day. On the 5^th^ day of life, five piglets of sow #872 received purified immunoglobulins. For that, each piglet was intraperitoneally administered 1 g purified immunoglobulin G (IgG) in 20 ml, derived from immune animals from a previous trial (File number: 7221.3-1.1-004/20).

On the 7^th^ day of life, all piglets of sows #17, #297, and #872 were oro-nasally inoculated with a spleen suspension containing 10^4.5^ HAD_50_ of the highly virulent ASFV strain ‘*Armenia2008*’. Each piglet received 0.5 ml in each nostril and 1 ml into the mouth. Clinical scores were assessed daily.

### 2.2 Viruses and cells

For oro-nasal inoculation of sows and piglets, porcine spleens, acquired during prior investigations on the pathogenesis of ASFV ‘*Estonia2014*’ or ASFV ‘*Armenia2008*’, were homogenized using sterile sea sand and subsequently titrated on monocytes/macrophages derived from peripheral blood mononuclear cells (PBMCs), as reported previously (23). For PBMC isolation, EDTA blood was obtained from healthy donor pigs housed within the FLI quarantine facility.

Cell extraction was accomplished by mixing whole blood with a 10% Hanks dextran solution (Sigma-Aldrich) at a volumetric ratio of 1:10. Following a 90 min incubation, the supernatant containing PBMCs was collected, along with erythrocytes that were diluted at a ratio of 1:10 with PBS, then stored at 4°C. The cells were subjected to washing and subsequently seeded at a density of 3 × 10^6^ cells/ml in Dulbecco′s Modified Eagle′s Medium (DMEM, supplemented with 10% fetal calf serum and 0.01% Penicillin/Streptomycin, Gibco) and incubated to perform hemadsorption tests (HATs), as described elsewhere (26). For 96-well plates, 3 × 10^5^ cells/well were seeded, for a 24-well plate 2.5 × 10^6^ cells/ml. All incubation steps were conducted at 37°C within a humidified environment and in the presence of 5% CO_2_ for a duration of 24 h. Recombinant colony-stimulating factor 2 (CSF2) was added at a concentration of 2 ng/ml after initial incubation.

The ASFV inoculum was thawed and titrated on differentiated macrophages in a 96-well plate. Erythrocytes were added to the cell medium at a ratio of 1:40, and the formation of rousettes assessed after 24 and 48 h. Prior to the oro-nasal inoculation, the virus stock was titrated on macrophages derived from PBMCs.

### 2.3 Sample collection and processing

Blood samples from all sows were collected prior to inoculation (0 dpi) to ensure absence of ASFV genome and antibodies. All surviving sows underwent regular testing for the presence of ASFV genome in blood, until a consistent negative result was observed across all subjects. Upon reaching the humane endpoint, blood, serum, and various organs, e.g., brain, bone marrow, spleen, and inguinal lymph nodes, were sampled. Blood samples from all piglets were collected on the 7^th^ day of life. For those piglets subjected to a challenge infection on the same day (born to sows #17, #297, #872), this time point served as control (0 dpi). Additionally, all piglets within the challenge group were sampled on the day of euthanasia (5–9 dpi). Blood, serum, and various organs, e.g., spleen, mandibular lymph node, gastrohepatic lymph node, liver, lung, kidney, and tonsil were collected and processed.

Blood and plasma samples were preserved at -80°C until further analysis. A pea-sized fragment from each organ was transferred to a 2 ml centrifugation tube containing 1 ml PBS and a 5 mm metal bead, followed by qPCR analysis after homogenization at 30 Hz for 3 min using a tissue lyzer (TissueLyzer II, Qiagen). Piglets of sows #984 and #986 remained without challenge infection and blood samples were taken weekly after birth to monitor kinetics of maternal antibodies. Ultimately, piglets of sow #984 were sampled on days 7, 10, 14, 21, 28, 35, 42, 49, and 60 after birth, whereas piglets of sow #986 were samples on days 7, 10, 14, 21, 28, 35, 46, and 53.

### 2.4 Nucleic acid extraction and RT-qPCR

In order to evaluate ASFV genomic loads in blood and various organs of all animals (with exception of piglets born to sows #984 and #986), 75 µl of whole blood or 100 µl of tissue homogenate underwent automated DNA extraction, using the NucleoMag^®^ VET Kit (Macherey-Nagel) on the KingFisher 96 Flex System (Thermo) in accordance with manufacturer’s instructions. Each extraction procedure included ASFV-negative pig’s serum to ensure the absence of contamination. Following extraction, qPCR was performed employing the virotype^®^ ASFV 2.0 PCR kit (Indical Bioscience). For quality assurance, the heterologous internal control provided by the manufacturer was included in all reactions. All qPCR reactions were carried out on a Bio-Rad C1000TM thermal cycler, equipped with the CFX96TM Real-Time System (Bio-Rad). Data visualization was performed with GraphPad Prism 9 (GraphPad Software Inc.).

### 2.5 Purification of immunoglobulins from sera of immune pigs

The serum for purification was derived from surviving pigs of a preceding trial (File number: 7221.3-1.1-004/20). The serum was tested positive for ASFV-p32 and ASFV-p72 antibodies, but negative for the presence of infectious ASFV particles or genome (qPCR negative). Approximately 1 L of serum was subjected to immunoglobulin purification. While kept on ice and under continuous agitation, ammonium sulfate [(NH4)_2_SO_4_] was added slowly to the serum, until a saturation of 50%. Following the addition, the solution was stirred for 1 h. Subsequently, the serum was centrifuged at 10,000 rpm at 4°C for 4 min and the supernatant was separated from the protein-containing pellet. The protein pellet was reconstituted in 1x PBS. Pre-equilibrated dialysis tubes were retrieved from the equilibration buffer, rinsed with ddH_2_O and sealed to one end. The dialysis tubes were filled with the ammonium sulfate-precipitated proteins and sealed at both ends. Dialysis was performed in two steps: initially, the tubes were incubated in 1x PBS on ice for 1 h. Thereafter, the tubes were transferred to a 4°C room and incubated in 1x PBS overnight under constant stirring. After incubation, the contents of the dialysis tubes were centrifuged at 4,000 × g at 4°C for 1 h. To mitigate the risk of anaphylactic reactions or other side effects in the piglets which received purified immunoglobulins, the entire solution containing the purified immunoglobulins was subjected to endotoxin removal. To ensure removal of >99% of endotoxins, Pierce™ High Capacity Endotoxin Removal Spin Columns (Thermo Fisher Scientific) were used following manufacturer’s instructions. The supernatant was stored at -80°C until further processing. An aliquot of the solution was used to assess the immunoglobulin concentration using a NanoDrop 2000c spectrophotometer (Thermo Fisher Scientific).

A total of 1 g (54.115 mg/ml) of purified immunoglobulin was administered intraperitoneally to five piglets from the naïve sow (#872).

### 2.6 Serology

To assess the kinetics of maternal antibodies in piglets with and without challenge infection, plasma samples were analyzed utilizing various methodologies conventionally employed for the detection of antibodies specific to ASFV antigens. Samples were examined using two complementary ELISA kits: (I) ID Screen^®^ African Swine Fever Competition (ID.vet), detecting antibodies directed against ASFV p32 and (II) Ingezim PPA COMPAC (Gold Standard Diagnostics), detecting antibodies specific to ASFV p72. The assays were conducted according to manufacturer’s instructions. Additionally, all plasma samples underwent semi-quantitative titration via immunoperoxidase tests (IPT). Given that the IPT is currently regarded as the most sensitive serological assay for identification of ASFV antibodies, the IPT results also functioned as reference for ELISA. Each plasma sample was titrated in the following dilutions: 1:40, 1:200, 1:1.000, 1:5,000, 1:25,000, 1:125,000, and 1:625,000.

### 2.7 Statistical analysis

Statistical analyses and data visualization were performed with GraphPad Prism 10.3.1 for Windows (GraphPad Software Inc., Boston, USA). Survival was analyzed by Log-rank (Mantel-Cox) test. Differences between multiple groups were assessed by ordinary one-way ANOVA with Holm-Šidák’s correction for multiple comparisons. Differences were considered significant if *P ≤* 0.05. Statistically significant differences are indicated as (*) *P ≤* 0.05, (**) *P ≤* 0.01, (***) *P ≤* 0.001, (****) *P ≤* 0.0001.

## 3 Results

### 3.1 Clinical manifestation of ASFV ‘*Estonia2014*’ in young sows

**Figure 2:**
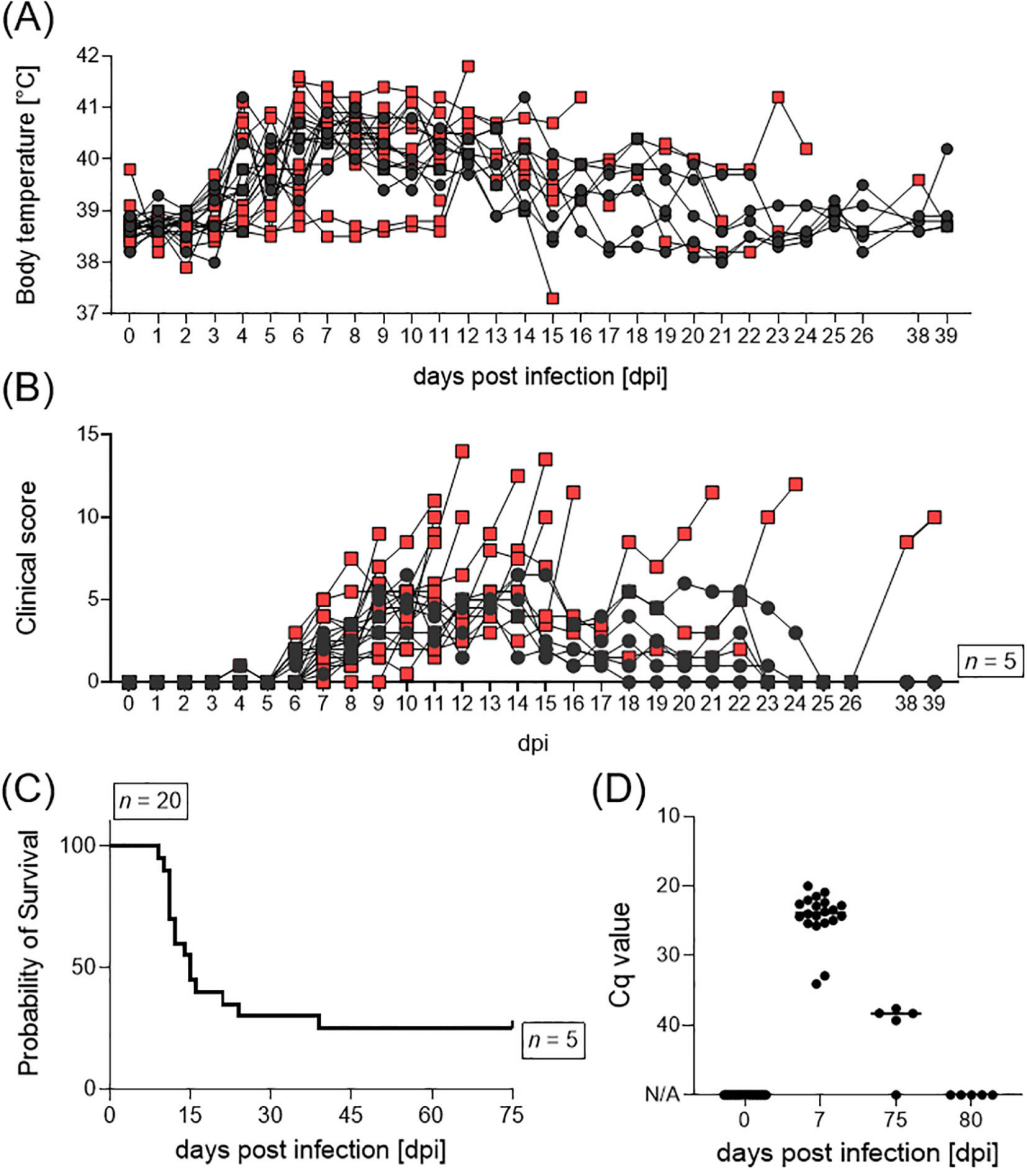
**(A)** Body temperature, **(B)** clinical score, **(C)** survival, and **(D)** ASFV genome loads in blood of female pigs upon inoculation with ASFV ‘*Estonia14*’. Animals which reached the humane endpoint are displayed as red squares, surviving animals as black dots.

All sows developed fever (≥ 40°C) after infection with the ASFV ‘*Estonia2014*’ strain, independent of having undergone oro-nasal inoculation or having contracted the virus through contact with infected conspecifics. The majority of animals developed fever at 4 dpi (**Figure 2A**). By day 13 pi, all surviving animals (#17, #297, #394, #984, #986) showed normal body temperatures. Clinical manifestation of ASFV infection was first observed at 4 dpi (**Figure 2B**). Most animals displayed diminished liveliness and reduced feed intake, alongside dermal and ocular erythema, as well as a change in gait and posture, indicative of pain and distress. Clinical manifestations and corresponding scores increased over time as most animals exhibited mild hemorrhages in the skin, significantly reduced liveliness and feed intake. Among the 15 sows that reached the humane endpoint, six exhibited pronounced neurological signs, e.g., paraplegia, total immobility of limbs, or seizures. Those individuals were euthanized prior to reaching a cumulative score of 15 points. Furthermore, one sow (#4) experienced acute hepatic failure at 39 dpi. Taken together, the ASFV ‘*Estonia2014*’ infection resulted in a survival rate of 20% over a 28-day span (**Figure 2C**). Macroscopic evaluations during necropsy of euthanized animals revealed lesions typical for ASFV infection, including hemorrhages in several organs, pulmonary edema, and pericardial effusion. Examination of spinal cords from animals with severe neurological signs revealed no apparent blockage of cerebrospinal fluid (e.g. due to abscess formation). However, these animals where highly positive in both qPCR and virus isolation of the following organs: cerebrum, cerebellum, brainstem, and meninges.

All sows underwent regular testing for the presence of ASFV genome in the blood, to determine the initiation point for synchronization. Only animals that tested negative for ASFV in qPCR were suitable for synchronization initiation. The animals were highly positive in blood at 7 dpi, all survivors tested negative at 80 dpi (**Figure 2D**). Moreover, blood and serum samples were collected during necropsy to determine the level of viraemia (**Supplementary Figure S1**). All animals, with the exception of the surviving sows (*n* = 5), were highly positive in blood on the day of necropsy. Additionally, sow #4, which ultimately succumbed to acute hepatic failure, tested positive for ASFV genome in blood, but not in serum. All sows, except surviving pigs and sow #4 (*n* = 6), exhibited high amounts of infectious ASFV particles in serum samples taken during necropsy.

### 3.2 Clinical manifestation of ASFV ‘*Armenia2008*’ in neonatal piglets

**Figure 3:**
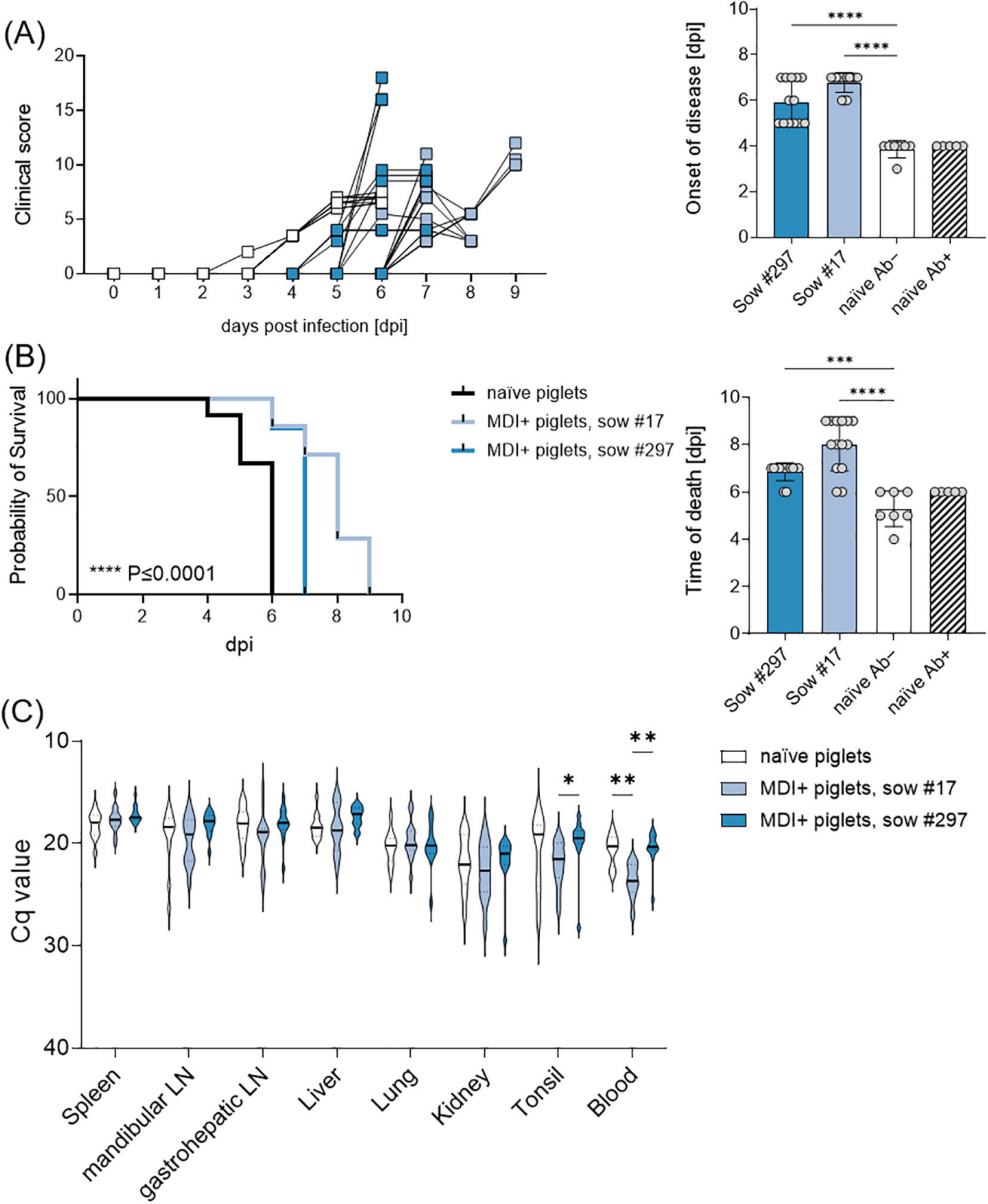
**(A)** Clinical score and summarized days of onset of disease, **(B)** survival and summarized days of euthanasia or death, and **(C)** viral genome loads in various organs and blood of piglets upon infection with ASFV ‘*Armenia08*’. Naïve piglets are shown separately in the summarized graphs based on whether they received passive immunity by antibodies (Ab+) or not (Ab−). Statistical significances were calculated by ordinary one-way ANOVA and Holm-Šidák’s correction for multiple comparisons. (*) *P ≤* 0.05, (**) *P ≤* 0.01, (***) *P ≤* 0.001, (****) *P ≤* 0.0001.

All piglets from sows #872, #17, and #297 were challenged with a highly virulent ASFV strain on their 7^th^ day of life. The piglets of the naïve sow #872, with no maternal-derived immunity (MDI, *n* = 12) and no purified immunoglobulins, served as infection controls (*n* = 7). All naïve piglets, irrespective of having received purified immunoglobulins or not, developed high fever (up to 42°C) and clinical signs such as diminished milk intake (almost no suckling acts) and reduced liveliness at day 4 pi (**Figure 3A**). Four individuals had to be euthanized at 4 dpi, while all remaining naïve piglets from sow #872 were euthanized on day 6 pi. To prevent the onset of mastitis, the sow was also euthanized on day 6 pi, exhibiting high loads of ASFV genome and infectious particles in inguinal lymph nodes, serum, tonsil, and spleen (**Figure 3C**; **Supplementary Table S2**).

Piglets from sows #17 (*n* = 14) and #297 (*n* = 13), which received MDI via colostrum ingestion, exhibited a delay of two days in the onset of clinical signs of infection relative to the MDI-negative piglets. The observed delay in onset of disease rendered highly significant (**Figure 3A**). However, despite this delay, all piglets ultimately developed clinical signs upon infection. Most piglets showed decreased liveliness, lack of interest in milk during maternal offering, skin hemorrhages, and diarrhea. Since most piglets refused to consume any milk (either maternal or substitute), they showed signs of malnutrition, e.g. retracted flanks and general weakness. Overall, all piglets of the naïve sow (#872) had to be euthanized by day 6 pi, piglets of sow #17 and #297 significantly later, by day 7 and 9 pi, respectively (**Figure 3B**). No significant differences were observed in ASFV genome loads in spleen, mandibular lymph node, gastrohepatic lymph node, liver, lung, kidney, or tonsil (**Figure 3C**). All piglets showed high amounts of infectious ASFV particles in the spleen. Pre-infected sows #17 and #297 were euthanized one day later and tested negative for ASFV genome and infectious particles in serum, spleen and inguinal lymph node.

### 3.3 Detection of maternal antibodies against ASFV

**Figure 4:**
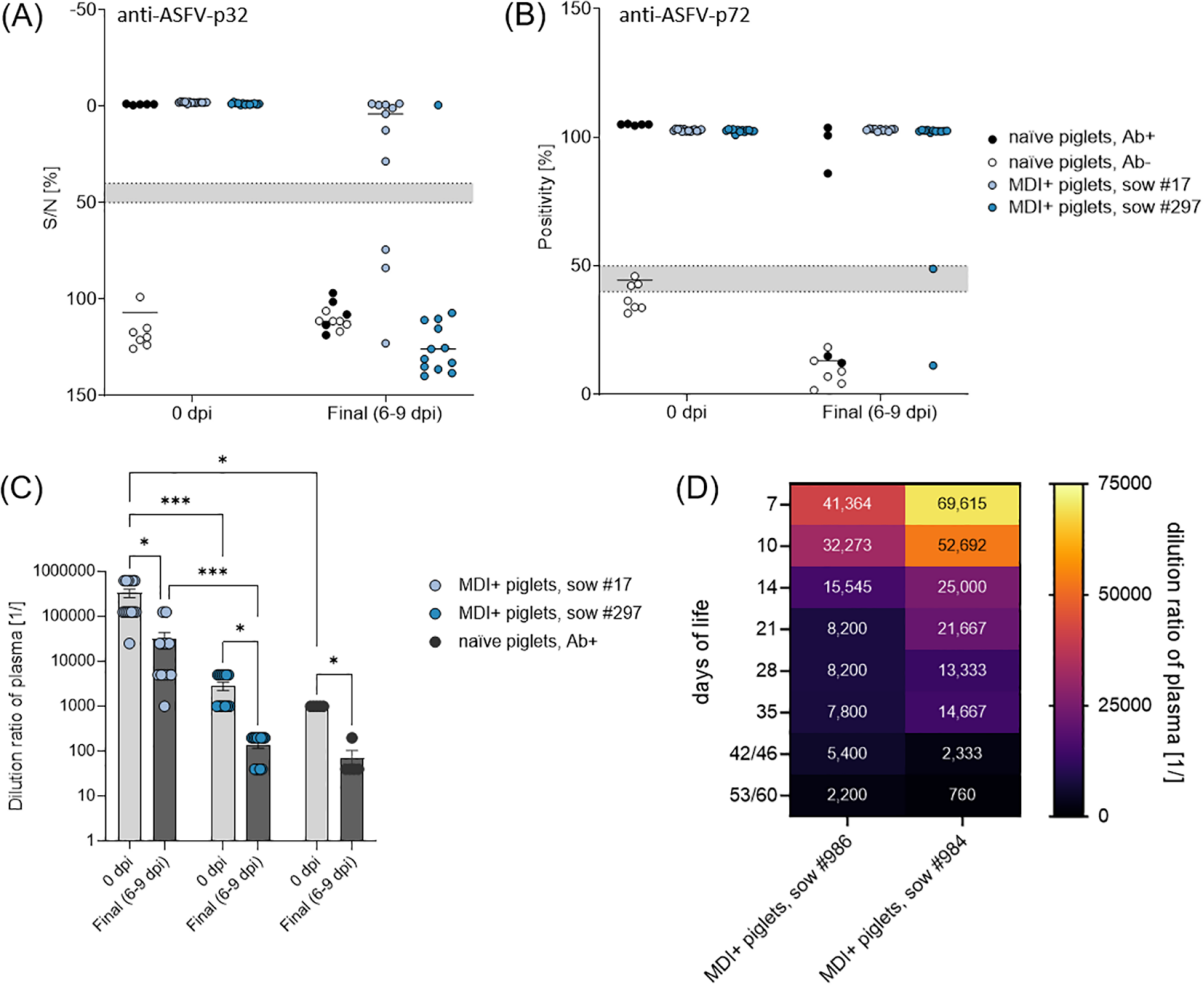
**(A)** Detection of anti-ASFV-p32 and **(B)** anti-ASFV-p72 antibodies in piglets prior and post challenge with ASFV ‘*Armenia08*’. **(C)** Total anti-ASFV antibody titers analyzed by IPT in challenged piglets 0 dpi and on the day of euthanasia (6–9 dpi). **(D)** Kinetics of maternal anti-ASFV antibodies in unchallenged piglets of sow #984 (*n* = 12) and #986 (*n* = 10) analyzed by IPT. Numbers in each field indicate mean dilution ratio. (*) *P ≤* 0.05, (***) *P ≤* 0.001.

To assess humoral immunity in piglets, we used two commercial ELISAs detecting antibodies against the early viral entry protein p32 (anti-ASFV-p32) and the late viral capsid protein p72 (anti-ASFV-p72). The passive transfer of 1 g purified immunoglobulins to piglets of naïve sow #872 (*n* = 5) resulted in successful detection of anti-ASFV-p32 and anti-ASFV-p72 antibodies prior to challenge infection at levels at the lower end of piglets with MDI. On the day of euthanasia or death, only three immunoglobulin-receiving piglets were still seropositive for ASFV-p72 antibodies, while all piglets tested negative for ASFV-p32 antibodies (**Figures 4A, B**). Naïve piglets without passively transferred immunoglobulins were seronegative prior and post challenge infection (**Figures 4A, B**).

All piglets from previously infected sows were positive for anti-ASFV-p32 and -p72 antibodies prior to challenge (**Figures 4A, B**). Interestingly, the duration of seropositivity was different in piglets of both sows after challenge. All piglets of sow #17 remained positive for anti-ASFV-p72 antibodies and only three piglets had lost reactivity against ASFV-p32 at the day of euthanasia. In contrast, only one piglet from sow #297 was still reactive against ASFV-p32 and two were already negative for anti-ASFV-p72 antibodies at the end of the trial (**Figures 4A, B**).

To further evaluate the quality of maternal antibodies or those acquired via transfer of purified immunoglobulins, all plasma samples were titrated in IPT. The last dilution steps at which plasma samples maintained positive reactivity are shown in **Figure 4C**. Piglets from sow #872 that received purified immunoglobulins showed a mean reactive dilution of 1:1,000, piglets of sows #17 and #297 showed 1:33,200 and 1:2,800, respectively. After challenge infection, all piglets demonstrated a noticeable reduction in antibody levels, with mean reactive dilutions of 1:70 for immunoglobulin receivers, and 1:31,000 and 1:140 for piglets of sows #17 and #297, respectively. Titers in piglets of sow #297 and the immunoglobulin receivers of sow #872 were largely comparable, whereas plasma of piglets of sow #17 seemingly contained much more ASFV-specific antibodies prior and post challenge infection.

Additionally, all piglets of sows #984 (*n* = 12) and #986 (*n* = 10) were sampled weekly after birth to monitor the dynamics of maternal ASFV-specific antibodies. All plasma samples displayed ~100% blocking in a commercial ASFV-p32-specific competitive ELISAs (**Supplementary Figure S2**). All plasma samples were further titrated in IPT, mean results of both litters are depicted in **Figure 4D**. Overall, all piglets showed high titers of ASFV-specific antibodies on the 7^th^ day of life with mean reactive titers of the litters of 1:150,000 (sow #984) and 1:42,000 (sow #986). In accordance with lower initial titers in piglets of sow #986, their titers declined faster than those of piglets of sow #984 over the study period (up to 60 days of life). Final mean titers were 1:2,200 and 1:760 in piglets of sows #986 and #984, respectively.

A summarized overview of the results involving neonatal piglets is shown in **Table 1**.

**Table 1:** Summarized results.

Sow	17	297	872	872	984	986
ASF status sow	immune	immune	naïve	naïve	immune	immune
Number of piglets	14	13	5 (Ab+)	7 (Ab -)	12	10
Group	Challenge	Challenge	Challenge	Challenge	Ab kinetic	Ab kinetic
Surviving piglets	0	0	0	0	N.a.	N.a.
Mean onset of disease [dpi]	6.8	5.9	4	3.9	N.a.	N.a.
Mean survival time [days]	8	6.8	6	5.3	N.a.	N.a.
Final Cq blood [Mean]	23.6	20.5	20	21	N.a.	N.a.
Ab titer [7th day of life]	332,143	2,846	1,000	N.a.	69,615	41,364
Ab titer [Final]	31,333	138	72	N.a.	760	2,200

Ab, antibody; N.a., Not applicable.

## 4 Discussion

This study aimed to elucidate the impact and dynamics of maternal immunity in the context of ASFV genotype II infection. Maternal antibodies have been extensively discussed as potential early modulators of ASFV infection and disease manifestation, with both beneficial and detrimental effects. In regions affected by ASF, maternal antibodies are an essential consideration when designing surveillance programs that include serological testing of young animals. In industrialized production settings, maternal immunity derived from field virus infections is unlikely to play a significant role, as all animals in affected herds (= epidemiological units) are typically culled. Conversely, in countries with backyard farming systems or in the absence of compensation schemes, surviving and valuable animals may be retained for breeding, potentially influencing infection dynamics. The same is true for wild suids in Europe and Asia.

We found no evidence of chronic or persistent infection in surviving sows. Although a slightly delayed onset of disease was noted, all piglets from immune sows exhibited acute disease courses upon highly virulent challenge infection and ultimately reached endpoint criteria. In principle, the delay in disease onset supports a role of MDI obtained via colostrum uptake, which includes both humoral and cellular immunity, and implies that humoral immunity by ASFV-specific antibodies alone is not sufficient to convey even partial protection. However, based on our and previous data, MDI shows limited capacity for protection against ASFV infections in piglets. Our observation that the presence of ASFV-specific antibodies alone is neither a correlate of protection nor a reliable indicator of any degree of protection confirm earlier studies (27–34).

Additionally, the monitoring of antibody titers in piglets born by immune mothers revealed a rapid decline in maternal ASFV-specific antibodies after challenge. Especially ASFV-p32-specific antibodies were fully depleted in piglets born by immune sow #297 or which received purified immunoglobulins. Interestingly, higher initial ASFV-specific antibody titers in piglets from sow #17 correlated with a pronounced delay in disease onset and slower antibody depletion after challenge. These findings further highlight the considerable individual differences in MDI, despite ultimately not affecting the fate of the piglets. It also demonstrated that only actively acquired humoral and cellular immunity, e.g., by vaccination, is able to protect animals from highly virulent ASFV infection. This observation should be included in future assessments regarding possible vaccine regimens and disease dynamics, not only for domestic pigs but also wild boar.

Antibody kinetics in piglets of immune sows #984 and #986 without challenge showed a similar, yet slower decrease in antibody titers. This is in line with previous results showing a half-life of colostrum-derived immunoglobulin of less than two months in piglets (35, 36), similar to humans (37). However, the half-life varies based on antigen-specificity and differences in the antibodies’ constant regions (38–40).

At least a part of the differences in antibody titers might be explained by the methodology in our study. Artificial insemination requires significant manipulation like medication, i.e., synthetic hormones like progesterone, to prepare and synchronize all sows for insemination and pregnancy. Importantly, among the known immunomodulating effects of progesterone are impairments of B cell and plasma cell maturation. We hypothesize that this might explain the considerable differences observed between piglets of sow #297 and sow #17. Sow #17 was part of the first inoculation group while sow #297 was part of the second, hence B cells of sow #17 had four additional weeks to mature before synchronisation with progesterone was initiated. Progesterone-mediated repression of B cell and antibody maturation, i.e., class switch recombination and somatic hypermutation, have already been shown in mice and humans (41, 42). However, a direct comparison of maternal antibody titers in piglets of all immune sows suggests that the amount of immunoglobulins and possibly reactive immune cells is also influenced by other factors. This explanation is further underscored by the fact that sows #17, #984, and #986 were all inoculated on the same day, but MDI (here: titers) differed considerably between the litters. Of note, the outcome and success of antibody responses are not only determined by simple amount but also by isotypes and differently executed effector functions (43), which were not part of this study.

Early studies conducted by Schlafer et al. in the 1980s were crucial for demonstrating the existence of MDI-mediated protection of piglets against ASFV, even after inoculation with very high virus doses. The work established that transfer of ASFV-specific antibodies, either through colostrum uptake or by administering purified immunoglobulins, offers partial protection of piglets from ASFV infection and disease manifestation (33). Similar to these findings, the present study observed a delay in the onset of clinical signs in piglets born by immune mothers. However, our results regarding the protective capacity of administered purified immunoglobulins are contrasting. We observed no delay in onset of clinical signs or severity in piglets which received purified immunoglobulins when compared to naïve, MDI-negative littermates. This was not a question of available amounts of immunoglobulins in the different piglets. Based on blood volume (44) and antibody amount in new-born piglets (45, 46), around 2–3 g of immunoglobulins are present in colostrum-fed individuals, which is comparable to the amount in piglets that received passive transfer of immunoglobulins in this study. This was also supported by the comparable results in antibody ELISAs and titration. Although the presence of passively transferred ASFV-p72- and ASFV-p32-targeting antibodies in those piglets prior to challenge was confirmed, they failed to offer even partial protection. While the titers of ASFV-targeting IgG were low in piglets that received purified immunoglobulins, it was comparable to titers of piglets born by sow #297, where a delay in onset of disease was observed in piglets. This suggests that the maternal leukocytes provided by colostrum from immune mothers took part in the immune response against ASFV but ultimately failed to protect against highly virulent ASFV. Based on the individual variances seen in humoral responses in this study (i.e., significant differences in titers between sows in this study), it can be assumed that transferred cellular responses would show similar variability. Hence, future research would benefit from studies which include an assessment of not only humoral immunity in general, but also immunoglobulin isotypes and antigen specificity of antibodies in the colostrum, as well as cellular immunity. In this context, challenge with lower doses and strains of lower virulence might be helpful.

Further differences between our and Schlafer’s studies may be attributed to two aspects: (I) the difference in virulence between the ASFV strains, comparing a moderately virulent genotype I isolate (47, 48) with a highly virulent isolate of genotype II or (II) changes in pig genetics over time. It was shown that genetically older breeds of various livestock species are more resilient in infection scenarios (49). For instance, older breeds of cattle and buffalo as a species are less susceptible for systemic diseases or associated febrile reactions and other clinical signs, e.g., foot-and-mouth disease for buffalo and brucellosis for cattle (49). For pigs, less data is available compared to ruminants, but breed-specific increases in resistance to porcine reproductive and respiratory syndrome virus 1 (PRRSV1) were reported (50). Moreover, pig breed has been shown to be a major influence of colostral IgA and IgG levels (51, 52), with older breeds, like Duroc, having higher colostral Ig levels than younger breeds like Landrace or Large-White (53, 54). However, our data also demonstrate considerable interindividual differences within the same breed and, thus, call for additional studies investigating the influence of maternal immunity against ASFV infections to account for the limited sample size and variability in our study.

While maternal immunity serves to protect the neonate during early life with an immunity that is specific for the environment the neonate will be born into (15), it also has some negative aspects. Especially antibodies have been shown to interfere with the efficacy of vaccination against several diseases in humans and various animals (55), predominately affecting live-attenuated and protein vaccines, for instance against measles virus, canine distemper virus (CDV) (18), feline herpesvirus 1 (FHV-1) (56), classical swine fever virus (CSFV) (57), and bovine viral diarrhea virus (BVDV) (58). Maternal immunity is the main cause of vaccine failure in puppies (18). Since all promising vaccine candidates against ASFV so far are live-attenuated viruses, vaccine strategies for very young animals need to be carefully assessed to avoid vaccinating animals too young and having maternal immunity interfere with vaccine efficacy. Nevertheless, our data imply the necessity of early active immunity in neonatal piglets against ASFV, underlining the need for safety and efficacy studies in all age groups.

Conclusively, our study provides further insights into the kinetics of maternal antibodies and overall robustness of maternal immunity during ASFV infection. Maternal antibodies were detected in piglets born to immune mothers over the whole observation period, but were not able to prevent lethal disease courses in piglets after a highly virulent challenge infection. Moreover, while these early antibodies are insufficient for protection and may falsely suggest protection, they might also impair vaccination efficiency. Critical interdependencies in vaccination of piglets complicates disease management and needs to be elucidated in future studies.

## Data Availability

The original contributions presented in the study are included in the article/**Supplementary Material**. Further inquiries can be directed to the corresponding author.
