# Supplementary material for: Maternal immunity and African swine fever virus: understanding the limits of passive protection
Source: Front Immunol. 2025 Jul 25;16:1593820. doi: 10.3389/fimmu.2025.1593820 (PMC12331693; doi:10.3389/fimmu.2025.1593820)
Supplement: Supplementary file 1 [file DataSheet1.docx]

|  | **sow ID #** | | | | |
| --- | --- | --- | --- | --- | --- |
|  | **17** | **297** | **872** | **984** | **986** |
| **piglet ID**  **#** | 1B | 1A | 4C | 1D | 8 |
|  | 2B | 2A | 5C | 2D | 10 |
|  | 3B | 3A | 6C | 4D | 11 |
|  | 4B | 4A | 7C | 5D | 12 |
|  | 5B | 5A | 8C | 6D | 13 |
|  | 6B | 6A | 10C | 7D | 14 |
|  | 7B | 8A | 13C | 8D | 16 |
|  | 8B | 9A | 14C | 9D | 17 |
|  | 9B | 10A | 15C | 10D | 18 |
|  | 10B | 11A | 18C | 11D | 19 |
|  | 11B | 12A | 19C | 12D | 20 |
|  | 12B | 13A | 20C | 13D |  |
|  | 13B | 14A |  | 14D |  |
|  | 14B |  |  |  |  |

**Supplementary Table 1.** Unique identifiers of all piglets born in this trial.

**Supplementary Table 2.** Virus isolation results of spleens of all challenged piglets.

| **MDI+ piglets, sow 17** | | **MDI+ piglets, sow 297** | | **MDI- piglets, sow 872** | |
| --- | --- | --- | --- | --- | --- |
| **Animal ID** | **Result** | **Animal ID** | **Result** | **Animal ID** | **Result** |
| 1B | ++ | 1A | ++ | 4C | ++ |
| 2B | ++ | 2A | ++ | 5C | ++ |
| 3B | ++ | 3A | ++ | 6C | ++ |
| 4B | ++ | 4A | ++ | 7C | ++ |
| 5B | ++ | 5A | ++ | 8C | ++ |
| 6B | ++ | 6A | ++ | 10C | ++ |
| 7B | ++ | 8A | ++ | 13C | ++ |
| 8B | ++ | 9A | ++ | 14C | ++ |
| 9B | ++ | 10A | ++ | 15C | ++ |
| 10B | ++ | 11A | ++ | 18C | ++ |
| 11B | ++ | 12A | ++ | 19C | ++ |
| 12B | ++ | 13A | ++ | 20C | ++ |
| 13B | ++ | 14A | ++ |  |  |
| 14B | ++ |  |  |  |  |

**Supplementary Figure 1.** qPCR results of blood and serum samples obtained from all sows at the day of euthanasia.

**Supplementary Figure 2.** Results of competitive ELISAs detecting ASFV-p72 and ASFV-p32 specific antibodies.
